# Social comparison and mental health among academics in Qatar: a cross-sectional study

**DOI:** 10.3389/fpsyg.2026.1732269

**Published:** 2026-03-24

**Authors:** Dalal Hammoudi Halat, Manar E. Abdel-Rahman, Ghadir Fakhri Al-Jayyousi, Ahmed Malki

**Affiliations:** 1 QU Health Office of Assessment and Accreditation, QU Health, Qatar University, Doha, Qatar; 2 Department of Public Health, College of Health Sciences, QU Health, Qatar University, Doha, Qatar; 3 Department of Biomedical Sciences, College of Health Sciences, QU Health, Qatar University, Doha, Qatar

**Keywords:** academic faculty, anxiety, burnout, depression, social comparison, stress

## Abstract

**Background:**

Social comparison is emerging as a prominent manifestation in today’s communities and can influence individuals’ wellbeing. This cross-sectional study aimed to assess social comparison among academic faculty in Qatar and explore associations with mental health indicators.

**Methods:**

The study used an online, anonymous cross-sectional survey conducted among academics in Qatar. Social comparison was assessed using the Iowa–Netherlands Comparison Orientation Measure (INCOM), while the Depression, Anxiety, Stress Scale-21 items (DASS-21) and Maslach Burnout inventory – Educators Survey (MBI-ES) were used to measure self-perceived depression, anxiety, stress, and burnout. Linear regression models and Modified Poisson regression models were used to assess multivariable relationships.

**Results:**

A total of 112 faculty responded to the survey, 54% of which were males and with average age of 44.2 ± 9.2 years. INCOM yielded a score of 26.5 ± 5.5, while self-perceived depression, anxiety, stress, scored at 9.4 ± 9.8, 11.9 ± 7.8, and 12.9 ± 10.5 using DASS-21. The burnout score using MBI-ES was 25 ± 18.8. In regression analysis, social comparison was significantly associated with depression (*p* = 0.006), stress (*p* = 0.001), and burnout (*p* = 0.002) but not with anxiety. Only social comparison of ability, but not opinion, correlated with mental health (*p* < 0.01).

**Conclusion:**

This preliminary report from Qatar regarding social comparison among academic faculty showed that it can play a role in evoking increased depression, stress and burnout. Further research is needed for better understanding of social comparison effects on the wellbeing of faculty.

## 1 Introduction

Social comparison, the tendency to use other people as sources of information to determine how we are doing, thinking, or behaving, has become an important source of competitive behavior and a central feature of human social life (Garcia et al., 2013; Cho and Ryu, 2016). Proposed by Leon Festinger in 1954 (Festinger, 1957), the social comparison theory explains how individuals determine their own worth, both personally and socially, based on the manner by which they compare to others in a variety of contexts, from daily social situations to organizational settings to economy. As such, individuals strive to improve their performance and simultaneously minimize discrepancies between their and other target persons’ level of performance, within a push to do better (Mussweiler et al., 2011). Social comparison could be a downward comparison, commonly known by contrasting oneself with others who are worse-off, motivated by an assumption to be a self-enhancement of wellbeing and self confidence (Stewart et al., 2013). On the other hand, social comparison could be an upward comparison, when individuals compare themselves with others whom they perceive to be better or more competent. This form of comparison can be motivating, as it sets for individuals higher standards to strive toward, inspiring them to improve their skills, achievements, or overall status (Caricati, 2012; Rahimi et al., 2017). However, upward social comparison can highlight perceived gaps between one’s self and superior others (Fleischmann et al., 2021), thereby generating negative emotions (Aubry et al., 2024).

According to Gibbons and Buunk (1999), the extent and frequency of comparison to others is different among individuals, reflecting a personal difference variable known as the social comparison orientation. For measurement and empirical testing of such individual dispositions to social comparison, Gibbons and Buunk developed a scale to measure differences in the tendency to make comparisons. The instrument, also known as the Iowa–Netherlands Comparison Orientation Measure (INCOM), measures the propensity toward engagement in social comparison by capturing aspects of one’s self and others, and consists of 11 core items assessed on a 5-point Likert scale. Higher scores indicate individuals to be more likely to compare themselves to others (Tigges, 2009). With original simultaneous development of both English and Dutch versions of INCOM, its reliability and validity were examined in several studies, showing that it is a valid and reliable tool to measure of social comparison orientation (Buunk et al., 2020). The INCOM has been well documented with multiple tests of construct and criterion-related validity (Tigges, 2009).

In terms of psychometric properties of the INCOM, Gibbons and Buunk, using exploratory principal components analyses, described two distinguishable factors within INCOM, of which one was labeled as ‘ability comparisons’ and the other as ‘opinion comparisons’ depending on specific statements used in the scale (Gibbons and Buunk, 1999). The distinction between ability- and opinion-oriented comparisons points out that opinions do not possess an objective basis of evaluation, whereas abilities are measurable in the form performance criteria. Therefore, social comparisons with others who are expected to be close to one’s own standing or position result in stable evaluations of both opinions and abilities; yet, abilities provoke an interest to become better, thus distinguishing them from opinions (Ozimek et al., 2023). It has been postulated that stronger ability-based social comparison decreases wellbeing via upward contrastive emotions, such depression and envy. By contrast, stronger opinion-based social comparison improves wellbeing via increased feelings of upward assimilative emotions, such as optimism and inspiration (Park and Baek, 2018).

Initial observations by Gibbons and Buunk showed that individuals with a high score on INCOM were better in terms of public and private self-consciousness, and may be worse in terms of self-esteem. Furthermore, they seek more comparisons, spend more time comparing themselves to others, and experience more powerful feelings as a result of this comparison. They show more interest in feelings and thoughts of others (Buunk and Gibbons, 2006). These observations were substantiated by more recent research indicating that social comparison orientation elicits negative emotions which could diminish self-esteem and psychological wellbeing (Lee, 2022). Nowadays, and with skyrocketing use of social media, social comparison opportunities and resulting negative outcomes continue to flourish. Evidence shows that frequent and more extreme upward comparisons resulted in immediate declines in one’s self-evaluation, as well as cumulative negative effects on individuals’ state self-esteem, trait self-esteem, mood, and life satisfaction (Midgley et al., 2021).

In terms of mental health, previous research has reported that social comparisons were related to higher depression and anxiety (Seabrook et al., 2016), and upward social comparison evoked career frustration (Fukubayashi and Fuji, 2021). Furthermore, significant positive correlations were found between upward social comparison and depression (Wang et al., 2020), sometimes with a gender difference inclined toward females (Weiguo et al., 2022), as well as among adolescents (Nesi and Prinstein, 2015). In an investigation done on nursing students during COVID-19, upward comparison explained 19% of depression among the participants (Kwak et al., 2022). Also, individuals engaging in comparison may experience stressful emotions, caused by the frustration or disappointment with themselves; they may also suffer from an increase in negative feelings, including depression and anxiety (Kulik and Gump, 1997). Unhealthy social comparison examined during COVID-19 in China predicted a higher level of stress (Yue et al., 2022), affecting wellbeing, growth and optimism (Yıldırım and Cicek, 2022). Moreover, strong links to high burnout were found in individuals with high social comparison orientation (Buunk and Brenninkmeijer, 2022). Buunk et al. (2010) demonstrated that social comparisons among nurses may predict future changes in their burnout levels. In fact, investigations of social comparison in relation with burnout also investigated the three burnout dimensions: emotional exhaustion (EE), commonly manifested as tiredness, deficient emotional energy, and problems in adaptation to work environment; depersonalization (DP), a set of negative behaviors, inappropriate attitudes, and suspicion in work; and low personal accomplishment (PA), labeled as low productivity, poor motivation, feelings of inadequacy, and uncertainty about one’s capacity to carry out essential tasks (Maslach et al., 1997), alongside low morale and an inability to deal with stressors (Yıldırım et al., 2021). Among teachers, high EE or DP had a positive and significant correlation with downward comparison (Saber Gigasari and Hassaskhah, 2017). Yet, in another investigation also among teachers, upward social comparison was positively correlated with EE (Hui et al., 2022). This finding for burnout was also replicated by Kitchel et al. (2012), who showed that teachers with higher social comparison attitudes are more prone to burnout, with the latter being identified as a predictor of quitting among teachers (Dilekçi et al., 2025). As such, findings regarding correlations between burnout and social comparison appear to be conflicting, and further focused insights are still needed. Such opposing findings have been attributed to several factors such as self-efficacy (Saber Gigasari and Hassaskhah, 2017), self-esteem, gender (Hui et al., 2022), and the nature of the cultural environment where social comparison manifests, in addition to personal relative perceptions of how individuals frame others as better off (upward comparison) or worse off (downward comparison) (Kitchel et al., 2012).

Among academic faculty, few studies have addressed the relationships between social comparison and mental health. For example, Yang and Robison (Yang and Robinson, 2018) showed that both upward and downward social comparison correlate with teaching anxiety and burnout. Furthermore, educators assessed by INCOM and who were negatively affected by social comparison suffered from distress and burnout, and needed counseling on how to use information from social comparison for self-esteem enhancement (Buunk and Brenninkmeijer, 2022). In academia, mental health concerns are on the rise, with increasing reports of stress, anxiety, depression and burnout (Johnson and Lester, 2022; Langley-Evans, 2023). The current context of global and competitive education is placing extremely demanding expectations on faculty. Teaching, supervising, serving the community, and securing research funds are only a few of the many duties that together overwhelm academics, affecting their mental health and wellbeing (Urbina-Garcia, 2020). The pressure faculty face to secure their career and navigate a highly competitive academic landscape, further augmented by attributes related to students, environmental constructs and organizational specifics, all have repercussions on their mental health (Hammoudi Halat et al., 2023). Previous research has shown links between such faculty mental health issues and social comparison (Buunk and Brenninkmeijer, 2022, Ramos Salazar et al., 2022).

As such, the conceptual framework for the present study hypothesizes that social comparison among academic faculty is associated with higher levels of mental issues that may include depression, stress, anxiety and burnout. This framework is grounded in Festinger’s social comparison theory and empirical findings, summarized above, linking social comparison with mental health in academic settings. Furthermore, the framework recognizes the conceptual basis for faculty, who routinely compare milestones such as research output, teaching evaluations, and academic career progression with colleagues perceived as being more successful, leading to emotional and psychological distress. The framework also appreciates that comparison is not uniform: it can be upward or downward, and is affected by individual and environmental characteristics within academia. To our knowledge, no previous studies in Qatar have investigated social comparison among academic faculty nor its effects on their wellbeing. The objectives of this study were to explore social comparison among faculty using the INCOM, and to determine associations between social comparison and its subcomponents of ability and opinion, and participant mental health attributes including depression, anxiety, stress and burnout.

## 2 Methods

### 2.1 Study design

The study population and methods were described previously (Hammoudi Halat et al., 2024). Briefly, we conducted a self-administered, cross-sectional questionnaire on all faculty at Qatar University (QU), the oldest and largest national institution of higher education in the State of Qatar (Al-Jayyousi et al., 2022), as part of a more comprehensive initiative that looked at various mental health, wellbeing, and social attributes among this population. The questionnaire was anonymous, voluntary, and completed online by consenting participants. The researchers conducted the investigation and reporting of this study according to guidelines set forth by the statement of Strengthening the Reporting of Observational Studies in Epidemiology (STROBE) (Cuschieri, 2019). The study was approved by Qatar University Institutional Review Board and received approval number QU-IRB 1900-E/23.

### 2.2 Study participants and procedures

All academic faculty across the 11 QU colleges were invited to respond to the questionnaire. Participation was voluntary, and no sampling procedure was applied; instead, the approach was a census invitation to the entire population of faculty members. This included those having various academic ranks, full- and part-time faculty, English and/or Arabic speakers, and those with or without academic administrator assignments. However, non-academic staff were excluded.

To collect data, the questionnaire was sent to QU emails of all faculty. For this purpose, an email announcement was prepared in Arabic and English and broadcasted to faculty, debriefing them about the study purpose, and inviting them to voluntarily respond to the questionnaire, reassuring about anonymity. Consent from each participant was obtained on the first questionnaire page. To engage more participants in the study and deal with non-response, the researchers were careful to clear and friendly survey design, and to ensure participants in the questionnaire invitation about the anonymity and confidentiality of responses. Moreover, two additional reminders about the questionnaire were sent over a two-month period, after which the questionnaire was locked.

### 2.3 Questionnaire design and outcome measures

Members of the research team created the study questionnaire in Microsoft Forms application, available through QU faculty portal. This application was used as faculty can access it only through institutional usernames and passwords, ensuring no participants from outside QU could access the questionnaire. Upon entry into Microsoft Forms, the questionnaire settings were adjusted so that any collection of names, email addresses, or personal identifies of participants were disabled, to ensure highest measures of privacy and data security. The questionnaire was first developed in English and then translated it to Arabic, whereby the latter version was intended to capture responses from faculty in the College of Sharia and Islamic Studies and the College of Law, who were predominantly Arabic-only speakers.

For the part of the project covered in the current study, the questionnaire consisted of 5 sections. In the first section, sociodemographic and academic data of faculty were collected, as well as their employment status and years spent at QU. Following this, another section addressed general lifestyle habits and inquired about any previous disease diagnosis (primarily chronic illnesses) and any previous mental health conditions.

Next, the questionnaire assessed self-perceived depression, anxiety and stress using the Depression, Anxiety, Stress Scale-21 items (DASS-21) (Tran et al., 2013). This standardized instrument provides measures of depression, stress, and anxiety and their severity using 21 items, 7 of which correspond to each mental condition. Of these items, each includes a statement and four short-response options from zero to 3. Participants were to asked to select how each statement applied to them over the previous week, with the following rating: zero (Never – Did not apply to me at all); 1 (Sometimes – Applied to me to some degree or some of the time); 2 (Often – Applied to me to a considerable degree or a good part of time); or 3 (Always – Applied to me very much or most of the time) (Henry and Crawford, 2005). Scores of the three conditions were calculated by summation of scores for the 7 items corresponding to each condition, and expressed as normal/mild and at least moderate at cut-off values of 0–13 for normal/mild and 14 + for at least moderate depression, 0–9 for normal/mild and 10 + for at least moderate anxiety, and 0–18 for normal/mild and 19 + for at least moderate stress. The DASS-21 was selected as it allowed to measure the three mental conditions in one tool, hence avoiding to further prolong the questionnaire. Furthermore, this tool has an accumulated body of evidence of validity and reliability across different settings to measure depression, stress, and anxiety, (Al-Kalbani et al., 2022; Laranjeira et al., 2023; Abdin et al., 2025) and also in the Arabic language (Moussa et al., 2017; Khalil et al., 2023; Al-Dassean and Murad, 2024). The validated Arabic translation of this scale was used in the Arabic questionnaire (Ali et al., 2021).

In another section of the questionnaire, faculty burnout was measured using the Maslach Burnout inventory – Educators Survey (MBI-ES). Developed by Maslach, Jackson, and Leiter (Maslach et al., 1997), the MBI has been described as the gold standard for assessing burnout in medical literature (Montero-Marín et al., 2012; El-Ibiary et al., 2017). Specifically, the MBI-ES was reported to be a reliable tool in educational settings (Kokkinos, 2006; Szigeti et al., 2017), including higher education (Kavanagh and Spiro, 2018). The survey is intended to discover how educators view their work and the individuals they work with closely. It uses the term “students” in many of the statements to showcase the educational role, and has been used to assess educators’ wellness in many languages (Hawrot and Koniewski, 2018; Kavanagh and Spiro, 2018; Vukmirovic et al., 2020) including Arabic (El Helou et al., 2016). This tool consists of 22 items of which 9 correspond to EE, 5 to DP, and 8 to PA. Faculty rated these items on a scale from zero to 6, whereby zero indicated that they never experienced the defined feeling in job, while the range from 1–6 indicated increased frequency of experiencing the feeling from 1 (a few times a year or less) to 6 (every day). A higher score indicated more burnout, and cutoffs for the three burnout dimensions were as follows: (i) for EE: Low ≤18, Moderate 19–26, and High ≥27; (ii) for DP: Low ≤5, Moderate 6–9, High ≥10; and (iii) for PA: Low ≥34, Moderate 29–33, High ≤28. For analysis done in this study, and according to previous literature (El-Ibiary et al., 2017; Szigeti et al., 2017), burnout in faculty was defined as scoring in the higher category of either EE or DP. As both the English and Arabic versions of MBS-ES are copyrighted, both were purchased from the copyright holder and were used in the study.

Institutional factors included in the analysis consisted of college affiliation, academic rank, years employed at QU, full- vs. part-time status, and whether the faculty member held an administrative role. Health-related variables included self-reported chronic conditions, sleep duration, physical activity, and smoking status. The main exposure variable in this study was social comparison, measured by the INCOM instrument in another section of the questionnaire. The 11 items addressing the two factors of “ability” and “opinion” were rated on a 5-point Likert scale. The ability factor, comprising 6 items, relates to performance using the terms “do,” “doing,” and “done.” As implied by its title, this factor assesses the tendency to compare to others in terms of abilities, by using statements like “I often compare myself with others with respect to what I have accomplished in life”. On the other hand, the second factor, labeled “opinions,” includes the remaining 5 items, none of which include the term “compare” or “comparison,” but are rather concerned with others’ thoughts or opinions, such as the statement “If I want to learn more about something, I try to find out what others think about it.” The total INCOM score was obtained by adding scores of the 11 items, and the scores of each of “ability” and “opinion” were computed by summation of the relevant 6 and 5 items, respectively. Two reverse-worded items, one in each factor, were reverse-coded before summation. Higher scores indicated a higher tendency for social comparison. The INCOM, together with the first two parts of the survey addressing demographics and health status of participants, were translated to Arabic by bilingual members of the research team fluent in both languages.

Following preparation of the questionnaire, and to ensure cultural appropriateness as well as linguistic and conceptual equivalence, the team collectively revised the survey with the Arabic translated parts. Then a piloting phase was conducted for the purpose of content validation and checking any ambiguities or misleading questions. A piloting sample of 5 faculty completed the questionnaire in each language, and provided their comments over content and clarity to the research team, who adjusted the questionnaires accordingly. Only minor linguistic adjustments were done to the demographics section of the English questionnaire, and to the demographics section and INCOM of the Arabic questionnaire. The remaining validated sections of the questionnaire were not modified. Piloting responses were excluded from results of the study.

### 2.4 Statistical methods

Data were summarized using percentages for categorical variables, and means and standard deviations (SD) or medians and interquartile ranges (IQR) for continuous variables. The t-test was used to assess differences between means. Normality assumptions were assessed by examining the distributions of continuous variables and by applying the Shapiro–Wilk *W* test.

Linear regression models and Modified Poisson regression models with a log link and robust variance estimation were used to assess crude and multivariable relationships for continuous and binary outcomes, respectively. Linear models assessed changes in continuous symptom scores, and modified Poisson models estimated prevalence ratios for clinically meaningful dichotomous outcome classifications (normal/mild vs. at least moderate). These methods allowed the examination of both symptom severity and clinically relevant cut-offs. The consistency of results across these methods supports the reliability of the observed associations. Multivariable associations were assessed to determine the effect of social comparison on depression, anxiety, stress, and burnout, adjusting for sociodemographic factors, university-related factors, and health-related factors across four models. Model 1, the crude model, included social comparison as the main exposure variable. Model 2 was the same as the crude model but further included sociodemographic factors. Model 3 included the variables in Model 2 and additionally accounted for university-related factors. Finally, Model 4 built upon Model 3 by incorporating health-related factors. All analyses were conducted with Stata, version 18.0.

Power calculations were performed at the end of the study, considering the correlation coefficients among depression, anxiety, Stress, burnout, and social comparison. Power was estimated assuming moderate correlation coefficients of 0.3 and 0.5. Given these coefficients, an effect size of 0.15, a Type I error rate of 0.05, and a total sample size of 112, the calculated power ranged from 45 to 66% for the current study, as previously reported (Hammoudi Halat et al., 2024). Sample size calculations were done using both Stata and G-power (Faul et al., 2009).

## 3 Results

Among our faculty, 63 responded to the Arabic questionnaire and 52 responded to the English questionnaire, out of which and 61 and 51 participants, respectively, gave their consent to participate, resulting in a total of 112 responses. This final number reflects those who voluntarily chose to complete the questionnaire. The majority were non-Qatari faculty (≈85%), with almost 35% aged 50 years and more, and slightly more than half of them were male participants (≈54%). Regarding university-related demographics, the number of participants from the College of Arts and Sciences and College of Engineering was the highest (≈30%); meanwhile, fewer participants were from health colleges (23.2%). The majority were PhD holders and full-time employees with 44.6% having been employed for more than 5 years and 57.1% not having administrative roles. In regard to health-related factors, slightly more than half of the participants reported sleeping at least 7 h per night, 48.2% perceived themselves to be active for at least 150 min per week, and the majority did not smoke. Moreover, the majority reported that they were not diagnosed with any chronic conditions (≈ 76%) nor mental health issues (86.6%). Details of participant demographic characteristics are shown in Supplementary material 1.

**Figure 1:**
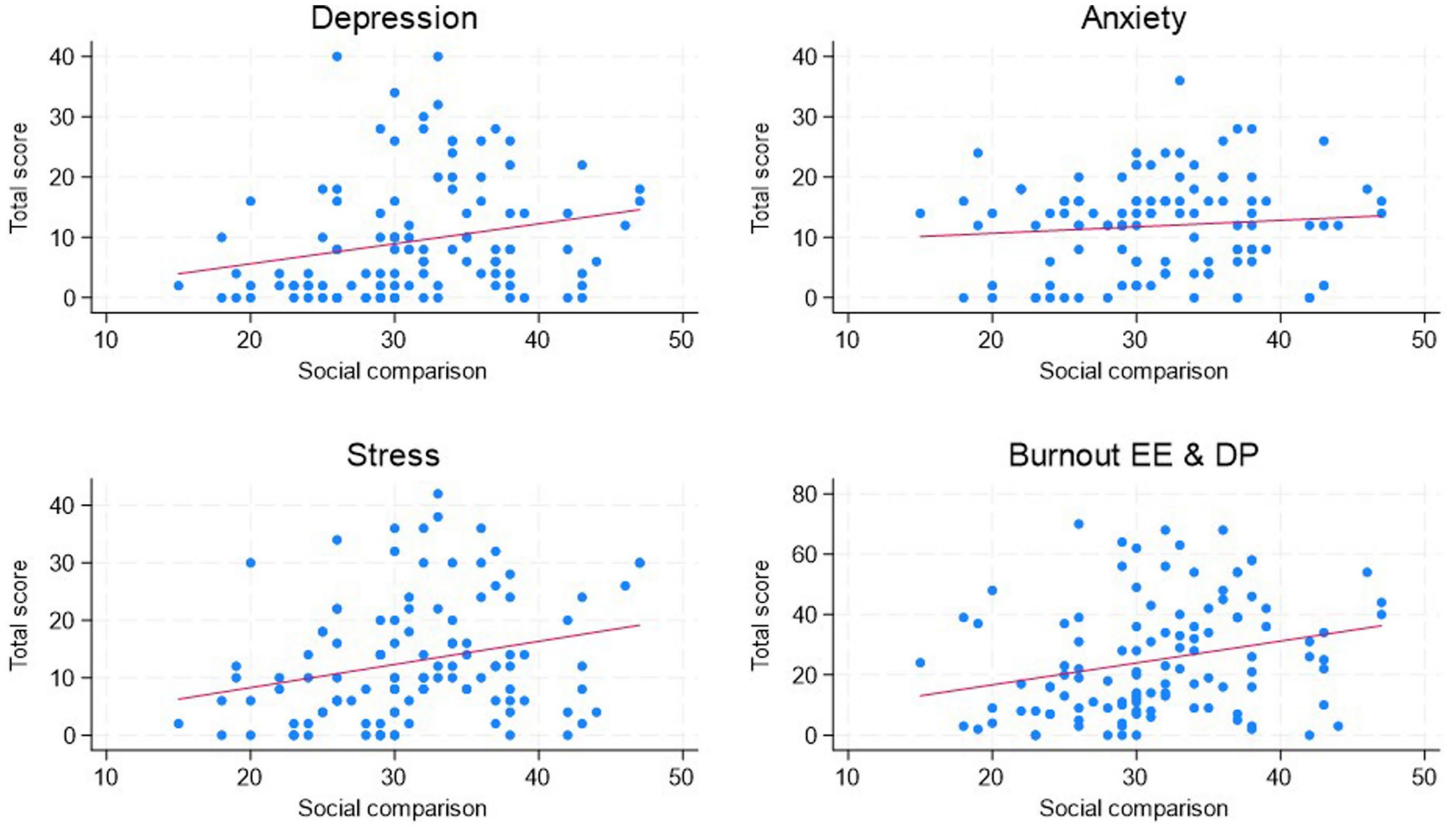
Scatter plots of depression, anxiety, stress, and burnout by social comparison. The solid red lines are fitted linear regression lines.

In the descriptive analyses, the total mean social comparison score was 26.5 ± 5.5, and the total mean depression, anxiety, stress, and burnout scores were 9.4 ± 9.8, 11.9 ± 7.8, 12.9 ± 10.5, and 25 ± 18.8, respectively (Table 1). Compared to total possible score, stress and burnout score almost at 30%, followed by anxiety and lastly depression. The correlations between the total social comparison score and depression, anxiety, stress, and burnout scores reported by the participants were also examined (Table 1). The results showed that social comparison was significantly correlated with depression (*r* = 0.23, *p* < 0.01), stress (*r* = 0.26, *p* < 0.01), and burnout (*r* = 0.26, *p* < 0.01), but not with anxiety (*r* = 0.09, *p* > 0.05) (Figure 1). The detailed correlation tables are shown in Supplementary material 2.

**Table 1:** Summary of the study descriptive data and correlation analysis (*n* = 112).

Total score	Depression	Anxiety	Stress	Burnout	Social comparison		
					Total	Abilities	Opinion
Depression	1.00						
Anxiety	0.53*	1.00					
Stress	0.82*	0.61*	1.00				
Burnout	0.60*	0.34*	0.67*	1.00			
SC	0.23*	0.09	0.26*	0.26*	1.00		
Abilities	0.27*	0.11	0.27*	0.32*	0.88*	1.00	
Opinion	0.09	0.04	0.15	0.09	0.79	0.42*	1.00
Number of items	7	7	7	14	11	6	5
Total possible score	42	42	42	84	55	30	25
Mean	9.4	11.9	12.9	25.0	26.5	13.87	12.63
SD	9.8	7.8	10.5	18.8	5.5	3.49	3.39
Cronbach’s α	0.91	0.64	0.90	0.92	0.79	0.81	0.66
Arabic questionnaire	0.92	0.83	0.92	0.92	0.81	0.84	0.68
English questionnaire	0.87	0.75	0.85	0.91	0.79	0.78	0.62

^*^p-value<0.01 (two-tailed). SC: Social Comparison, SD: Standard Deviation.

However, when examining the correlations between the two components of social comparison and the different mental health issues, the results showed that the “abilities” component was significantly correlated with depression, stress, and burnout (*p* < 0.01); meanwhile, the “opinion” component did not show any significant correlations with the reported mental health issues.

Linear regression was conducted to examine relationships between social comparison scores of the participants and mental health scores (Table 2). For each score increase in social comparison, after adjusting for sociodemographic factors, university-related factors, and health-related factors, there was 0.30 (95% CI 0.01,0.59, *p* < 0.1) increase in depression score. Social comparison did not show any significant association with anxiety even after the adjustment for the different independent variables. In addition, for each score increase in social comparison, after adjusting for the mentioned independent variables, there was 0.44 (95% CI 0.14,0.73, *p* < 0.05) and 0.93 (95% CI 0.39,1.46, *p* < 0.05) increase in stress and burnout scores. The results showed a slight difference in the depression, stress, and burnout scores across the four models.

**Table 2:** Linear regression results for the total social comparison score.

	Model 1 (crude) Beta [95% CI]	Model 2 Beta [95% CI]	Model 3 Beta [95% CI]	Model 4 Beta [95% CI]
Depression	0.33^**^ [0.06,0.60]	0.30^**^ [0.03,0.57]	0.31^**^ [0.03,0.59]	0.30^**^ [0.01,0.59]
*R*-squared	0.05	0.11	0.21	0.26
Adjusted *R*-squared	0.04	0.06	0.09	0.10
Anxiety	0.11 [−0.11,0.32]	0.12 [−0.09,0.33]	0.09 [−0.10,0.29]	0.09 [−0.11,0.30]
*R*-squared	0.01	0.15	0.36	0.4
Adjusted *R*-squared	0.00	0.10	0.27	0.28
Stress	0.40^***^ [0.12,0.69]	0.37^**^ [0.09,0.66]	0.43^***^ [0.13,0.72]	0.44^***^ [0.14,0.73]
*R*-squared	0.07	0.15	0.25	0.32
Adjusted *R*-squared	0.06	0.1	0.14	0.18
Burnout (EE and DP)	0.73^***^ [0.21,1.24]	0.71^***^ [0.22,1.21]	0.88^***^ [0.38,1.38]	0.93^***^ [0.39,1.46]
*R*-squared	0.07	0.18	0.30	0.31
Adjusted *R*-squared	0.06	0.14	0.20	0.17

***p < 0.01, **p < 0.05, *p < 0.1. Model 1 (crude): Social comparison, Model 2: Crude model + Sociodemographic factors, Model 3: Model 2 + university-related factors, Model 4: Model 3 + health-related factors.

The bivariate analysis to examine the association of depression, anxiety, stress, and burnout (treated as binary) by social comparison (treated as continuous) was also conducted (Table 3). Participants who reported “at least moderate” level of depression were more likely to report higher social comparison mean score (33.8 ± 6.0) compared to those who reported “Normal/ Mild” depression (*p* = 0.013). Moreover, for participants who reported “at least moderate” level of stress and answered “yes” for burnout (EE High or DP High), these were more likely to report higher social comparison mean score compared to participants who did not report this level of stress or burnout (*p* = 0.008, *p* = 0.011, respectively).

**Table 3:** Bivariate analysis of depression, anxiety, stress, and burnout by social comparison.

	Social comparison					
	Overall scale		Abilities subscale		Opinion subscale	
	Mean (SD)	*p*-value	Mean (SD)	*p*-value	Mean (SD)	*p*-value
Depression		0.013		<0.001		0.587
Normal/Mild (0–13)	30.4 (6.8)		13.3 (4.3)		17.1 (3.7)	
At least ‘moderate’ (14+)	33.8 (6.0)		16.4 (4.4)		17.5 (2.9)	
Anxiety		0.942		0.662		0.474
Normal/ Mild (0–9)	31.5 (6.6)		14.0 (4.1)		17.5 (3.5)	
At least ‘moderate’ (10+)	31.4 (6.9)		14.4 (4.8)		17.0 (3.5)	
Stress		0.008		0.001		0.357
Normal/ Mild (0–18)	30.4 (6.6)		13.4 (4.3)		17.0 (3.5)	
At least ‘moderate’ (19+)	34.2 (6.5)		16.6 (4.5)		17.7 (3.2)	
Burnout (EE High or DP High)		0.011		0.003		0.261
No	30.1 (6.5)		13.2 (4.3)		15.8 (4.5)	
Yes	33.4 (6.6)		16.9 (3.7)		17.6 (3.0)	

Furthermore, multivariable analysis using Modified Poisson regression models showed a significant association between social comparison and mental health issues (depression, stress, and burnout) among participants in the crude and adjusted prevalence ratio (PR) (Table 4). After adjusting for sociodemographic factors, university-related factors, and health-related factors (Model 4), results showed that for every score increase in social comparison, the prevalence of depression increased by 5% (*p* = 0.006), the prevalence of stress increased by 8% (*p* = 0.001), and the prevalence of burnout increased by 5% (*p* = 0.002).

**Table 4:** Crude and adjusted prevalence ratio (PR) of depression, anxiety, stress, and burnout for the total social comparison score.

	Crude model 1		Model 2		Model 3		Model 4	
	PR [95% CI]	*p*-value	aPR [95% CI]	*p*-value	aPR [95% CI]	*p*-value	aPR [95% CI]	*p*-value
Depression	1.05*** [1.02,1.10]	0.005	1.05** [1.01,1.09]	0.015	1.05*** [1.01,1.09]	0.008	1.05*** [1.02,1.09]	0.006
Anxiety	1.00 [0.98,1.02]	0.941	1.00 [0.98,1.02]	0.961	1.00 [0.98,1.01]	0.621	1.00 [0.98,1.02]	0.852
Stress	1.06*** [1.02,1.11]	0.002	1.06*** [1.02,1.10]	0.006	1.07*** [1.02,1.12]	0.004	1.08*** [1.03,1.12]	0.001
Burnout	1.05*** [1.01,1.08]	0.008	1.04** [1.01,1.08]	0.014	1.05*** [1.01,1.09]	0.009	1.05*** [1.02,1.09]	0.002

Crude model 1: Social comparison, Model 2: crude model + Sociodemographic factors, Model 3: Model 2 + university-related factors, Model 4: Model 3 + health-related factors. ***p < 0.01, **p-value <0.05, *p-value < 0.1.

Linear regression was conducted to examine relationships between the ability component score of social comparison and mental health scores of participants (Table 5). For each score increase in social comparison, after adjusting for sociodemographic factors, university-related factors, and health-related factors, there was 0.60 (95% CI 0.18,1.02, *p* < 0.01) increase in depression score. Social comparison of ability, however, did not show any significant association with anxiety even after the adjustment for the different independent variables. In addition, for each score increase in social comparison, after adjusting for the mentioned independent variables, there was 0.73 (95% CI 0.29,1.16, *p* < 0.01) and 1.71 (95% CI 0.94,2.47, *p* < 0.01) increase in stress and burnout scores, respectively.

**Table 5:** Linear regression results for the ability social comparison score.

	**Model 1 (crude)** **Beta [95% CI]**	**Model 2** **Beta [95% CI]**	**Model 3** **Beta [95% CI]**	**Model 4** **Beta [95% CI]**
Depression	0.59*** [0.19,0.98]	0.59*** [0.20,0.98]	0.60*** [0.20,1.01]	0.60*** [0.18,1.02]
*R*-squared	0.07	0.14	0.24	0.29
Adjust *R*-squared	0.06	0.09	0.12	0.14
Anxiety	0.19 [−0.13,0.51]	0.21 [−0.09,0.52]	0.19 [−0.10,0.48]	0.19 [−0.12,0.49]
R-squared	0.01	0.15	0.36	0.41
Adjust R-squared	0.00	0.10	0.27	0.28
Stress	0.63*** [0.21,1.05]	0.64*** [0.22,1.05]	0.70*** [0.27,1.12]	0.73*** [0.29,1.16]
*R*-squared	0.07	0.16	0.26	0.34
Adjust *R*-squared	0.07	0.12	0.16	0.2
Burnout (EE and DP)	1.32*** [0.58,2.07]	1.39*** [0.68,2.10]	1.59*** [0.87,2.31]	1.71*** [0.94,2.47]
*R*-squared	0.10	0.23	0.34	0.36
Adjust *R*-squared	0.09	0.19	0.25	0.23

***p < 0.01, **p < 0.05, *p < 0.1. Model 1(crude): Social comparison, Model 2: Crude model + Sociodemographic factors, Model 3: Model 2 + university-related factors, Model 4: Model 3 + health-related factors.

Furthermore, multivariable analysis using Modified Poisson regression models showed a significant association between social comparison and mental health issues (depression, stress, and burnout) among participants in the adjusted prevalence ratio (PR) (Table 6). After adjusting for sociodemographic factors, university-related factors, and health-related factors (Model 4), results showed that for every score increase in ability component of social comparison, the prevalence of depression increased by 10% (<0.001), the prevalence of stress increased by 14% (<0.001), and the prevalence of burnout increased by 9% (*p* = 0.001).

**Table 6:** Crude and adjusted prevalence ratio (PR) of depression, anxiety, stress, and burnout for the ability social comparison score.

	Crude model 1		Model 2		Model 3		Model 4	
	aPR [95% CI]	*p*-value	aPR [95% CI]	*p*-value	aPR [95% CI]	*p*-value	aPR [95% CI]	*p*-value
Depression	1.12*** [1.05,1.19]	0.001	1.11*** [1.04,1.18]	0.002	1.10*** [1.04,1.17]	0.001	1.10*** [1.04,1.16]	<0.001
Anxiety	1.01 [0.98,1.04]	0.648	1.01 [0.98,1.04]	0.581	1.01 [0.98,1.03]	0.689	1.01 [0.98,1.04]	0.485
Stress	1.13*** [1.05,1.21]	0.001	1.12*** [1.04,1.20]	0.002	1.13*** [1.05,1.21]	0.001	1.14*** [1.07,1.21]	<0.001
Burnout	1.08*** [1.03,1.14]	0.003	1.08*** [1.03,1.14]	0.003	1.09*** [1.03,1.15]	0.003	1.09*** [1.04,1.15]	0.001

Crude model 1: Social comparison, Model 2: crude model 1 + Sociodemographic factors, Model 3: Model 2 + university-related factors, Model 4: Model 3 + health-related factors. ***p < 0.01, **p-value < 0.05, *p-value < 0.1.

Regarding the opinion component of social comparison, and after adjusting for sociodemographic factors, university-related factors, and health-related factors, the results showed no significant association with any of the mental health issues (Table 7). Furthermore, multivariable analysis using Modified Poisson regression did not show any significant associations between this component and mental health issues (Table 8). Supplementary material 3 includes figures showing the correlations of social comparison of both abilities and opinions with depression, anxiety, stress, and burnout scores.

**Table 7:** Linear regression results for the opinion social comparison score.

	Model 1 (crude) Beta [95% CI]	Model 2 Beta [95% CI]	Model 3 Beta [95% CI]	Model 4 Beta [95% CI]
Depression	0.25 [−0.28,0.79]	0.13 [−0.42,0.67]	0.13 [−0.42,0.69]	0.07 [−0.49,0.64]
*R*-squared	0.01	0.07	0.17	0.22
Adjust *R*-squared	0.00	0.01	0.05	0.06
Anxiety	0.08 [−0.34,0.51]	0.09 [−0.32,0.50]	0.03 [−0.35,0.42]	0.03 [−0.36,0.42]
*R*-squared	0	0.14	0.35	0.4
Adjust *R*-squared	−0.01	0.09	0.26	0.27
Stress	0.45 [−0.12,1.02]	0.32 [−0.25,0.89]	0.40 [−0.19,0.98]	0.39 [−0.20,0.97]
*R*-squared	0.02	0.10	0.20	0.27
Adjust *R*-squared	0.01	0.05	0.08	0.12
Burnout (EE and DP)	0.49 [−0.54,1.51]	0.30 [−0.71,1.31]	0.57 [−0.46,1.60]	0.53 [−0.55,1.61]
*R*-squared	0.01	0.12	0.22	0.23
Adjust *R*-squared	0.00	0.07	0.11	0.07

***p < 0.01, **p < 0.05, *p < 0.1. Model 1(crude): Social comparison, Model 2: Crude model + Sociodemographic factors, Model 3: Model 2 + university-related factors, Model 4: Model 3 + health-related factors.

**Table 8:** Crude and adjusted prevalence ratio (PR) of depression, anxiety, stress, and burnout for the opinion social comparison score.

	Crude model 1		Model 2		Model 3		Model 4	
	aPR [95% CI]	*p*-value	aPR [95% CI]	*p*-value	aPR [95% CI]	*p*-value	aPR [95% CI]	*p*-value
Depression	1.02 [0.95,1.10]	0.545	1.00 [0.93,1.08]	0.936	1.01 [0.93,1.10]	0.78	1.01 [0.93,1.09]	0.878
Anxiety	0.98 [0.95,1.03]	0.472	0.98 [0.95,1.02]	0.41	0.97 [0.94,1.01]	0.104	0.97 [0.94,1.01]	0.153
Stress	1.04 [0.96,1.14]	0.324	1.03 [0.95,1.11]	0.536	1.04 [0.94,1.14]	0.457	1.04 [0.93,1.17]	0.487
Burnout	1.04 [0.97,1.11]	0.237	1.03 [0.97,1.10]	0.382	1.04 [0.98,1.11]	0.227	1.04 [0.98,1.11]	0.217

Crude model 1: Social comparison, Model 2: crude model + Sociodemographic factors, Model 3: Model 2 + university-related factors, Model 4: Model 3 + health-related factors. ***p < 0.01, **p-value <0.05, *p-value<0.1.

## 4 Discussion

The current study represents a snapshot into the attribute of social comparison among academic faculty, and its relation to mental status, for the first time in Qatar. The key findings of the current investigation reveal average levels of social comparison as revealed by scores on the INCOM and both of its factors relative to the total possible score. While the INCOM higher scores indicated higher tendency to social comparison without definitive interpretation of scores in the original instrument, our results reveal lower scores than those reported in literature among educators (Saber Gigasari and Hassaskhah, 2017), as well as among the adult population in different countries (Buunk et al., 2020; Schneider et al., 2025), indicating contextual and cultural differences with our surveyed population. Moreover, and with different correlation models, social comparison significantly correlated with faculty depression, stress, and burnout, whereby the prevalence of these conditions increased between 5 and 8% with every score increase in social comparison, and results were significant for the abilities component. Our results provide partial support for the initial hypothesis and underscore the impactful role of social comparison in shaping faculty members’ psychological wellbeing within the competitive academic environment. Such findings expose, for the first time in Qatar, social comparison as a noteworthy construct of the academic career, and link it to faculty mental conditions. With the opinion component of social comparison being associated with depression, stress, and burnout, it may be inferred that comparisons rooted in skills, performance and competence tend to affect mental conditions, rather than comparison of opinions or beliefs, which do not directly affect one’s sense of success or achievement. Yet, the relatively small sample size may have reduced the statistical power to detect significant associations for anxiety, which should be interpreted with caution. In addition to the effect of small sample, our questionnaire included a self-assessed anxiety measure not specifically focused on teaching anxiety (Ramos Salazar et al., 2022), and this could have buffered or masked the association between social comparison and anxiety in our context of faculty. Possible feelings of security as to holding a PhD degree and being employed for more than 5 years at QU may also have made most of our participants interpret others’ success as an inspiration, reducing anxiety even when comparison is frequent. This remains elusive, yet an interesting area to explore. Given the unique stressors associated with a job in higher education, and the multiple responsibilities often juggled by faculty (Hammoudi Halat et al., 2023), social comparison may add to their behaviors to obtain, measure, or act upon similarities or contrasts between themselves and others (Tor and Garcia, 2023). Social comparison among faculty should be critically viewed in light of recent reports of its effect on individuals’ perceptions and emotions regarding their careers, resulting in career frustration (Fukubayashi and Fuji, 2021). Like for other individuals, competitive judgments and decisions by faculty can be informed by social comparison, which offers information about an individual’s relative position, abilities, and achievements, resulting in possible potential advantages, yet in possible behavioral effects (Tor and Garcia, 2023).

The correlations of social comparison depicted in this study have been described elsewhere. In a study of faculty during COVID-19, it was concluded that job burnout among faculty was correlated positively with social comparison, and had an effect on teaching satisfaction (Ramos Salazar et al., 2022). As reported by Steers and Colleagues (Steers et al., 2014), social comparison impacts psychological health and increases depressive symptoms. Also, particularly among females and adolescents, technology-based social comparison and seeking of feedback were associated with depression (Nesi and Prinstein, 2015), while reducing social comparison behavior may alleviate depressive symptoms (Aubry et al., 2024). Furthermore, and similar to our findings, Yue and Colleagues (Yue et al., 2022) reported that more social comparison is associated with higher levels of perceived stress. As such, our results add to the accumulating data about relations between social comparison and burnout, depression, and stress. Additional and more focused research on social comparison among academic faculty in relation to their specific career roles and their mental wellbeing are tempting to further explore.

Regarding burnout, our results showed that either high EE or high DP correlated with higher scores on social comparison, again for the abilities component. As reported by Han and Colleagues (Han et al., 2020), feelings of EE and reduced perception of accomplishment leading to burnout mostly emerge from comparisons with other colleagues in similar conditions. Moreover, in comparison to colleagues who were promoted, the more the similarity and the wider the gap, the more likely for employees to experience burnout (Zhao et al., 2017). A previous study on teachers found that those with more downward comparison experience more exhaustion and isolation from students and other aspects of the job, that is higher EE and DP. Contrary to this, PA indicated a positive correlation with upward comparison, whereby teachers who compare themselves to those whose performance is better experience higher levels of PA and less burnout (Saber Gigasari and Hassaskhah, 2017). Taken together, these data contribute to understanding of how social comparison affects EE and DP, and perhaps provide insights for applicable interventional studies that assess approaches to protect faculty mental health in academic settings.

We further tested two distinct associations of social comparison factors with mental conditions: social comparison of ability and that of opinion. Splitting these two components is essential because, while the ability factor involves outcomes depicted as performance, growth and accomplishments, the opinion factor involves presenting or sharing individual values and beliefs. According to literature, the ability factor might evoke more negative emotions such as jealousy, compared to the opinion factor that evokes relatively less negative emotions (Steinberger and Kim, 2023). In our correlation analysis, only the ability factor impacted more depression, stress and burnout, indicating that our participants may be more focused on comparing their achievements to those of others rather than comparing their thoughts or beliefs, and this factor exerted more consequences on their wellbeing. Such distinct relations call for ongoing research that can involve qualitative assays for elaboration of social comparison experiences, as well as comparative studies looking at social comparison in various academic contexts and its effect on the mental status of faculty in different communities, universities, and specialties.

## 5 Strengths, limitations, and recommendations for future research

The strength of this study depends upon using validated, well-documented scales to assess faculty self-perceptions of their social comparison attitudes as well as their mental health. Also, it is the first study on a national level that digs into the relationships among these constructs, laying the ground for a more wholistic view of mental health and wellbeing among this population. We assessed faculty from all specialties, and included a wide array of academic ranks and careers, as well as opinions reflective of both the English and the Arabic versions of our research instrument, thereby generating a reasonably representative proportion of academic faculty at our institution. We also considered different models in the analysis to establish robustness in the results. However, limitations to this research do exist. The total number of faculty at QU during the year data were collected was about 1,300, so that the modest sample size, corresponding to those who voluntarily chose to complete the questionnaire after its initial launching and two reminders, may indicate that the results may not be representative of QU faculty population. The calculated post-hoc power of 45–66% is relatively low and is acknowledged. This limitation reflects the voluntary nature of survey participation, which resulted in a smaller than anticipated sample size. As such, while the findings provide important information, they should be interpreted with caution due to the limited statistical power. Future studies that are more inclusive of faculty members are, therefore, recommended for future research on social comparison and mental health. The questionnaire was self-administered, raising the possibility that recall bias and subjective perceptions play a role in the findings. With voluntary participation, and with our somehow lengthy instrument, we expect that some participants did not complete the questionnaire till the end, making us lose some responses and thereby some impact on the study results. The study was conducted in the single public higher education institution in the country, and it would be promising, in upcoming research, to capture multi-center data including private institutions as well. The demographic distribution including 85% of non-Qatari faculty may have influenced our findings, as most of the responses came from this population, so the results may not fully represent the views of the smaller group of Qatari faculty. Finally, the cross-sectional design allowed us to capture social comparison and mental health attributes at one point in time; perhaps longitudinal analysis done at different points in time to test the effect of faculty-specific interventions on social comparison and wellbeing, are interesting to further explore. The model of burnout was based on EE and DP, and future investigations considering the role of PA as well are recommended to get deeper and newer insights.

## 6 Conclusion

In conclusion, our study revealed social comparison aspects among faculty, and founded an understanding of how these significantly correlate with mental health conditions. With academia being a profession in which burnout and mental issues may be prevalent, ample opportunity to compare one’s self with colleagues exists. In light of this, a novel dimension of this research is to further describe and look at social comparison among academic faculty. Interventions to understand how social comparison can affect faculty behavior in pursuit of their goals and achieving career success, are warranted. Furthermore, checking if these interventions may predict changes in stress, depression, and burnout among academic faculty are welcoming to further study.

## Data Availability

The datasets for this study can be requested from corresponding authors upon a reasonable request.
