# Supplementary material for: Social comparison and mental health among academics in Qatar: a cross-sectional study
Source: Front Psychol. 2026 Mar 24;17:1732269. doi: 10.3389/fpsyg.2026.1732269 (PMC13055615; doi:10.3389/fpsyg.2026.1732269)
Supplement: Supplementary file 1 [file Supplementary_file_1.docx]

Supplementary Material

# Supplementary Material 1

# Participants’ sociodemographic characteristics (n=112)

|  | **n (%)** |
| --- | --- |
| *Sociodemographic factors* |  |
| **Age in years, mean (SD)** | 44.2 (9.2) |
| **Age in years^^^** |  |
| <30 | 6 (5.4) |
| 30-39 | 30 (26.8) |
| 40-49 | 36 (32.1) |
| 50+ | 39 (34.8) |
| **Gender** |  |
| Female | 52 (46.4) |
| Male | 60 (53.6) |
| **Nationality** |  |
| Non-Qatari | 95 (84.8) |
| Qatari | 17 (15.2) |
| *University-related factors* |  |
| **College affiliated with** |  |
| Arts and Science, Engineering | 32 (28.6) |
| Humanities | 29 (25.9) |
| Health-related | 26 (23.2) |
| Other colleges | 25 (22.3) |
| **Highest academic degree** |  |
| BSc/ MSc | 27 (24.1) |
| PhD | 85 (75.9) |
| **Administrative role** |  |
| No | 64 (57.1) |
| Yes | 48 (42.9) |
| **Work duration at QU in years** |  |
| <=2 | 31 (27.7) |
| >2 to 5 | 31 (27.7) |
| 5+ | 50 (44.6) |
| **Employment type** |  |
| Full-time | 103 (92.0) |
| Part-time | 9 (8.0) |
| *Health-related factors* |  |
| **Sleep, at least 7 hours per night** |  |
| No | 55 (49.1) |
| Yes | 57 (50.9) |
| **Physical activity, at least 150 minutes per week** |  |
| No | 58 (51.8) |
| Yes | 54 (48.2) |
| **Smoking, cigarettes/ vape/ nargileh** |  |
| No | 103 (92.0) |
| Yes | 9 (8.0) |
| **Medical diagnosis** ^&^ |  |
| No | 85 (75.9) |
| Yes | 27 (24.1) |
| **Mental health diagnosis** ^&&^ |  |
| No | 97 (86.6) |
| Yes | 15 (13.4) |

# ^^^ 1 missing observations

# ^&^ Includes hypertension, heart disease, diabetes, dyslipidemia, chronic kidney disease, chronic lung disease, or cancer.

# ^&&^ Includes schizophrenia, panic attacks, bipolar disorder, eating disorders, or others.

# Supplementary Material 2

Spearman correlation matrix between mental health outcomes, social comparison measures, and sociodemographic/work-related factors (n = 112)

|  | **Depression total score** | **Anxiety total score** | **Stress total score** | **Burnout EE & DP total score** | **Social comparison** | **Social comparison of abilities** | **Social comparison of opinions** | **Age in years** | **Gender** | **Nationality** | **College affiliated with** | **Highest academic degree** | **Hold any administrative role?** | **Duration at QU in years** | **Employment type** |
| --- | --- | --- | --- | --- | --- | --- | --- | --- | --- | --- | --- | --- | --- | --- | --- |
|  |  |  |  |  |  |  |  |  |  |  |  |  |  |  |  |
| **Depression total score** | 1 |  |  |  |  |  |  |  |  |  |  |  |  |  |  |
| **Anxiety total score** | 0.5157* | 1 |  |  |  |  |  |  |  |  |  |  |  |  |  |
| **Stress total score** | 0.7902* | 0.5928* | 1 |  |  |  |  |  |  |  |  |  |  |  |  |
| **Burnout EE & DP total score** | 0.6217* | 0.3252* | 0.6884* | 1 |  |  |  |  |  |  |  |  |  |  |  |
| **Social comparison score** | 0.3082* | 0.0982 | 0.2796* | 0.3058* | 1 |  |  |  |  |  |  |  |  |  |  |
| **Social comparison of abilities** | 0.3563* | 0.1183 | 0.2738* | 0.3350* | 0.8999* | 1 |  |  |  |  |  |  |  |  |  |
| **Social comparison of opinions** | 0.1136 | 0.0282 | 0.1675 | 0.1238 | 0.7493* | 0.4167* | 1 |  |  |  |  |  |  |  |  |
| **Age in years** | -0.1715 | -0.177 | -0.1253 | -0.2953* | -0.0446 | 0.0091 | -0.1291 | 1 |  |  |  |  |  |  |  |
| **Gender** | -0.2386* | 0.0485 | -0.2702* | -0.2363* | -0.0965 | -0.0156 | -0.1894* | 0.2067* | 1 |  |  |  |  |  |  |
| **Nationality** | 0.064 | 0.2299* | 0.0976 | 0.0216 | 0.0605 | 0.0386 | 0.0874 | -0.2600* | -0.2049* | 1 |  |  |  |  |  |
| **College affiliated with** | -0.0394 | -0.0986 | -0.0374 | 0.0452 | -0.1256 | -0.1028 | -0.123 | 0.1186 | 0.0203 | 0.0115 | 1 |  |  |  |  |
| **Highest academic degrees** | -0.1256 | -0.0484 | -0.112 | -0.0717 | 0.0611 | 0.0894 | -0.0607 | 0.2889* | 0.3961* | 0.0057 | 0.1665 | 1 |  |  |  |
| **Hold any administrative role?** | 0.0239 | 0.0017 | 0.0907 | 0.115 | -0.0713 | -0.0652 | -0.0569 | -0.0869 | -0.062 | 0.0862 | 0.0482 | -0.1024 | 1 |  |  |
| **Duration at QU in years** | 0.1732 | 0.2468* | 0.2616* | 0.1318 | -0.0036 | 0.0357 | -0.0271 | 0.2826* | -0.0134 | 0.0652 | -0.0753 | 0.0558 | 0.129 | 1 |  |
| **Employment type** | 0.1899* | 0.0751 | 0.0586 | 0.0081 | 0.0829 | 0.08 | 0.0903 | -0.3680* | -0.12 | -0.0335 | -0.0788 | -0.4477* | -0.1233 | -0.2959* | 1 |

*Significant at 0.05

Spearman correlation matrix between mental health outcomes, social comparison measures, and health-related factors (n = 112)

|  | **Depression total score** | **Anxiety total score** | **Stress total score** | **Burnout EE & DP total score** | **Social comparison** | **Social comparison of abilities** | **Social comparison of opinions** | **Sleep** | **Physical activity** | **Mental health disorder** |
| --- | --- | --- | --- | --- | --- | --- | --- | --- | --- | --- |
|  |  |  |  |  |  |  |  |  |  |  |
| **Depression total score** | 1 |  |  |  |  |  |  |  |  |  |
| **Anxiety total score** | 0.5157* | 1 |  |  |  |  |  |  |  |  |
| **Stress total score** | 0.7902* | 0.5928* | 1 |  |  |  |  |  |  |  |
| **Burnout EE & DP total score** | 0.6217* | 0.3252* | 0.6884* | 1 |  |  |  |  |  |  |
| **Social comparison** | 0.3082* | 0.0982 | 0.2796* | 0.3058* | 1 |  |  |  |  |  |
| **Social comparison of abilities** | 0.3563* | 0.1183 | 0.2738* | 0.3350* | 0.8999* | 1 |  |  |  |  |
| **Social comparison of opinions** | 0.1136 | 0.0282 | 0.1675 | 0.1238 | 0.7493* | 0.4167* | 1 |  |  |  |
| **Sleep** | -0.1876* | -0.0389 | -0.1861* | -0.1464 | -0.2203* | -0.1793 | -0.2101* | 1 |  |  |
| **Physical activity** | -0.1635 | -0.1923* | -0.2311* | -0.1026 | -0.0338 | -0.0341 | 0.0325 | 0.2330* | 1 |  |
| **Mental health disorder** | 0.0797 | 0.026 | 0.1088 | -0.0801 | -0.0078 | 0.035 | -0.0428 | 0.0943 | 0.041 | 1 |

# Supplementary Material 3

Correlation of social comparison of abilities with depression, anxiety, stress, and burnout scores

Correlation of social comparison of opinions with depression, anxiety, stress, and burnout scores
